# Assessing the performance of different irrigation systems on lettuce (*Lactuca sativa* L.) in the greenhouse

**DOI:** 10.1371/journal.pone.0209329

**Published:** 2019-02-04

**Authors:** Zijing Chen, Yingyan Han, Kang Ning, Chen Luo, Wei Sheng, Shenglin Wang, Shuangxi Fan, Yanfang Wang, Qian Wang

**Affiliations:** 1 Department of Vegetable Sciences, Beijing Key Laboratory of Growth and Developmental Regulation for Protected Vegetable Crops, China Agricultural University, Beijing, China; 2 State Key Laboratory of Crop Biology, Scientific Observing and Experimental Station of Environment Controlled Agricultural Engineering in Huanghuaihai Region, Ministry of Agriculture/Shan Dong Agricultural University, Taian, Shandong, China; 3 Plant Science and Technology College, Beijing University of Agriculture, Beijing, China; 4 Beijing Agriculture technology promotion station, Beijing, China; Huazhong Agriculture University, CHINA

## Abstract

Lettuce (*Lactuca sativa* L.) is a very important leafy vegetable in China and is commonly grown using furrow irrigation. In order to improve production efficiency, greenhouse experiments were conducted at Experimental Station, China Agricultural University, Beijing, China using furrow irrigation (FI), micro-sprinkler irrigation (MS), plastic film mulching irrigation (PF) and a combined plastic film mulching–micro-sprinkler irrigation system (PF+MS) to study their effects on soil physical characteristics, water distribution, root morpho-physiological traits, nutrition absorption, lettuce yield and water use efficiency for a spring crop and autumn crop in 2015 (Fig 1). Root length, root surface area, and root density were significantly higher under PF and PF+MS than under FI. Moreover, these traits were higher under MS than under FI but these differences were not significant. The soluble protein, soluble sugar, and Vitamin C content of lettuce decreased in the order PF+MS > PF > MS > FI in both crops. In the spring crop, the biological yield of MS, PF, and PF+MS was 7.22%、36.77%、43.20% higher than FI, respectively. In the spring crop, biological water use efficiency (BWUE) of FI, MS, PF and PF+MS was 20.93, 25.24, 36.81 and 38.54 kg m^−3^, respectively. The BWUE of MS, PF, and PF+MS was 20.59%, 75.87% and 84.14% higher than FI. Economic water use efficiency (EWUE) of FI, MS, PF and PF+MS was 17.06, 21.31, 31.11 and 32.31 kg m^−3^, respectively. The EWUE of MS, PF, and PF+MS was 24.91%, 82.36% and 89.39% higher than FI, respectively. The autumn crop achieved a higher WUE than the spring crop. The results suggested that the combined plastic film mulching-micro-sprinkler irrigation was the most suitable irrigation approach for increasing lettuce yield.

**Fig 1:**
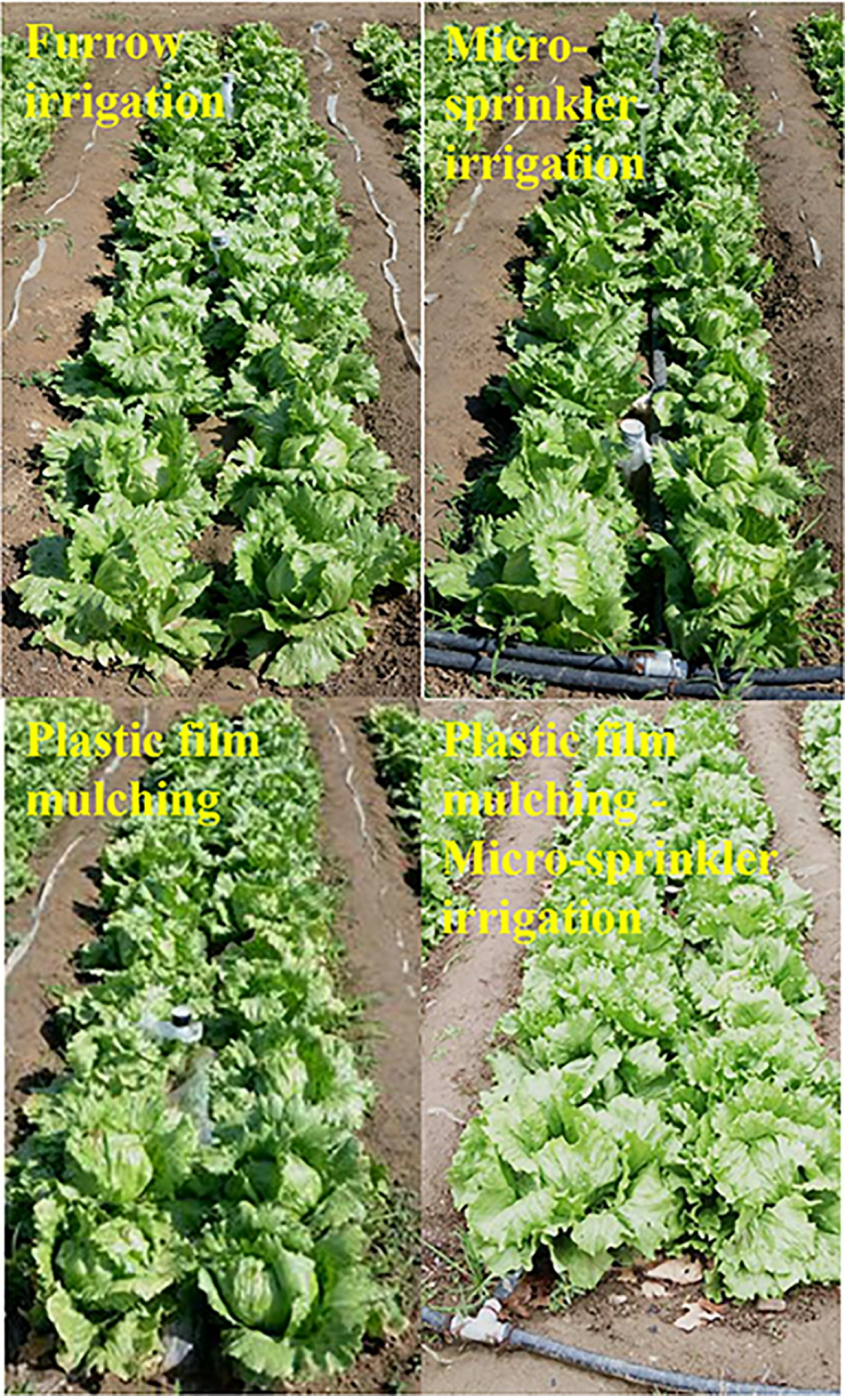
Illustration of different irrigation systems used for this study.

## Introduction

Lettuce (*Lactuca sativa* L.) is one of the most significant leafy vegetables that planted worldwide and consumed throughout the year. The world yield of lettuce and chicory was 24896 tons in 2013 (http://faostat3.fao.org/browse/Q/QC/E). Lettuce is rich in health-promoting compounds such as vitamins C and E, lutein, polyphenols and fibers [1], which plays an important role in avoiding the incidence of many chronic diseases [2]. Water scarcity is an increasingly serious phenomenon, especially in tropic and inland region. Moreover, water supply for agriculture is often insufficient owing to the increasing human demand. In light of the increasing attention on the utilization of scarce water resources, it is necessary to improve water use efficiency (WUE) [3, 4].Improving irrigation efficiency could enhance water management [5].Yield of vegetable crops in greenhouses has increased largely over recent decades. In addition, increased greenhouse vegetable yield has lower environmental impact compared with field cultivation [6].Under greenhouse conditions, measuring the status of the soil water using sensors alongside the selection of objective irrigation methods according to real-time measurements is necessary [7]. Appropriate irrigation methods and water-saving irrigation technologies are required to help manage the shortage of irrigation water.

Irrigation systems can directly influence crop performance and result in qualitative and quantitative improvements in vegetable yield [8]. Under-irrigation usually leads to reduced crop yield and quality, whereas over-irrigation results in water loss, increased vulnerability to disease and environmental pollution owing to fertilizer loss [9]. Therefore, efficient irrigation systems are needed not only to reduce environmental pollination but also to promote sustainable utilization of resources [10]. Common irrigation systems include furrow irrigation, micro-sprinkler irrigation, plastic film mulching irrigation and drip irrigation. Of these, furrow irrigation is the traditional irrigation system in China. Unfortunately, the irrigation amount cannot be easily controlled in this approach and alternate irrigation methods might be required to improve the IWUE [11]. Micro-irrigation can accurately regulate the irrigation amount and is popular in vegetable, orchards and wide-row planted crops [12–14]. Plastic film mulching can save water, change the soil temperature [15] and promote plant growth [16]. Drip irrigation can save water and increase crop production by transferring small amounts of water frequently to the periphery of the roots of plants [17]. In addition, combining drip irrigation with plastic film mulch is useful for increasing yield for cotton and potato [18–20].However, irrigation technology combining Micro-sprinkler irrigation and Plastic film mulching is rarely used.

To establish a better irrigation system, it is necessary to improve our understanding of lettuce root growth, the spatial distribution of roots in the soil and their relation with yield under different irrigation methods. Roots play an important role in absorbing water and nutrients [21].Hodgson found that more roots develop under a drip irrigation system than under furrow irrigation at the early boll filling stage [22].Mulch drip irrigation methods could dramatically promote root growth in the upper soil profile.

The conservation of irrigation water is becoming increasingly important in lettuce yield to reach optimal yield due to water scarcity, especially in Beijing, which has a massive human population. To our knowledge, there are few studies on the effect of different irrigation systems in lettuce production. Given the above considerations, the aim of this study was to explore the impact of different water-saving irrigation systems on soil physical characteristics, water distribution, plant height, root morpho-physiological traits, nutrition absorption, lettuce yield and WUE.

## Materials and methods

### Experimental site and growing condition of lettuce

The study was carried out from January 2015 to October 2015 in the greenhouse at the Experimental Station, China Agricultural University, Beijing (39°54N’, 116°23E’), China. The ‘sheshou 101’ variety was chosen for experiment. The spring crop is sowed in the 27^th^ January, planted in the 28^th^ March (five-leaf seedling) and harvested in the 17^th^ May. The autumn crop is sowed in the 16^th^ July, planted in the 11^th^ August (five-leaf seedling) and harvested in the 30^th^ September.

Seeds of lettuce were sown in a 50-hole tray filled with a mixture of peat substrates and vermiculite (v:v = 2:1), adding 10% volume chicken manure compost. The lettuce seedlings were planted in two rows in each furrow with a 30 cm row space× 50 cm line space. The furrow was 1.00 m in width and 3.60 m in length. Before planting, 3000 kg of chicken manure compost and 185 kg of compound fertilizer were added to the soil. In 3000 kg chicken manure compost, the whole amount of N,P,K is more than 4% and the whole amount of organic matter is more than 30%. In 185 kg compound fertilizer, N: P_2_O_5_: K_2_O = 15: 10: 15,the amount of total nutrient is more than 51%.Detailed soil physical characteristics before autumn crop were showed in Table 1: soil nutrient contents were 2.41% for organic matter, 1.572 g kg^−1^ for total N, 169.46 mg kg^−1^ for available N, 188.57 mg kg^−1^ for available P and 53.65 mg kg^−1^ for available K [23].The soil volume weight was 1.16 g cm^−3^ and the loam soil had an average field capacity at 32% (cm^3^ cm^−3^).The field of spring crop was close to that of autumn crop and the soil physical characteristics were similar.

**Table 1:** Soil physical and chemical properties in the greenhouse.

	Organic mater(%)	Total N(g·kg^-1^)	Available N(mg·kg^-1^)	Available P (mg·kg^-1^)	Available K(mg·kg^-1^)	Volume weight(g·cm^-3^)	Field capacity(cm^3^·cm^-3^)
Autumn crop	2.41	1.572	169.46	188.57	53.65	1.16	32.0

### Treatments for irrigation systems and irrigation amounts

Four irrigation systems were examined in this study: furrow irrigation (FI), micro-sprinkler irrigation (MS), plastic film mulching (PF) and plastic film mulching combined with micro-sprinkler irrigation (PF+MS). Of these, FI is the local irrigation method widely used by the local farmers and thus we treated FI as the control. Three replicates (furrows) were conducted for each treatment. The MS irrigation system had a main rigid pipe with a diameter of 50 mm and micro-sprinkler pipe with a diameter of 28.6 mm and 0.1 mm holes on the pipe. The pipes were placed between the two rows. The PF irrigation system was implemented using common white agricultural mulching films. The PF+MS irrigation system placed the MS pipes under the plastic film mulching.

### Environmental conditions measurement

Daily environmental conditions in the greenhouse, including air temperature, relative humidity and light intensity were recorded with a “Greenhouse Baby” (Xinyuan company, Beijing, China). The Greenhouse Baby has a real-time monitoring function to help farmers conduct the production management through simulated voice. The Greenhouse Baby controlling relative humidity and light intensity is placed 150 cm above the ground. The temperature sensor controlling the Greenhouse Baby was placed 100 cm above the ground. The sensor collected data every 60 min, recorded data in the machine and copied to the computer. Environmental condition data of different growth stages in greenhouse are shown in S1 Table.

### Soil sample collection, soil bulk intensity

Before planting, soil samples from 0–10, 10–20 and 20–30cm soil depth were collected using the soil-drilling method in an S-shape in five-spot pattern for the measurement of basic soil indexes in March and August, 2015. After harvest, soil samples from 0–10, 10–20 and 20–30 cm were collected using the same method. To improve efficiency and veracity of soil sampling in testing bulk density, the cutting ring method was used. Five drills in diagonal in every treatment were collected. Three layers of soil sample were mixed for measurement of soil bulk intensity.

### Soil pH, electrical conductivity (EC), mineral element and temperature analysis

For pH analysis, 4 g of air-dried soil and 10 ml of distilled water were mixed and left to stand for 30 min. The pH of the soil suspension was measured using a pH meter (Delta 320, Shanghai, China), with three replicates for every treatment. For EC analysis, 8 g air-dried soil and 10 ml of distilled water were mixed and left to stand for 30 min. Then the EC of soil suspension was measured with an EC meter (DDS.307A, Shanghai, China).

Total N content in soils was measured with a semi-micro-open method [24]. Soil available K content was measured using 1.0 mol L^−1^ NH4OAc*-*extracted flame photometry [25]. Soil available P content in soils was measured by 0.5 mol L^−1^ NH4OAc*-*extreacted molybdenum antimony spectrophotometry [26].

Soil temperature was measured by the Greenhouse Baby. The temperature probes were placed 10 and 20 cm below the soil surface. Data were recorded every 60 min.

Irrigation water distribution pattern measurement

### Soil water content, Irrigation content (I, m^3^), storage water content(△W,m^3^), evaporation content (E, m^3^), transpiration content (T, m^3^)

**Fig 2:**
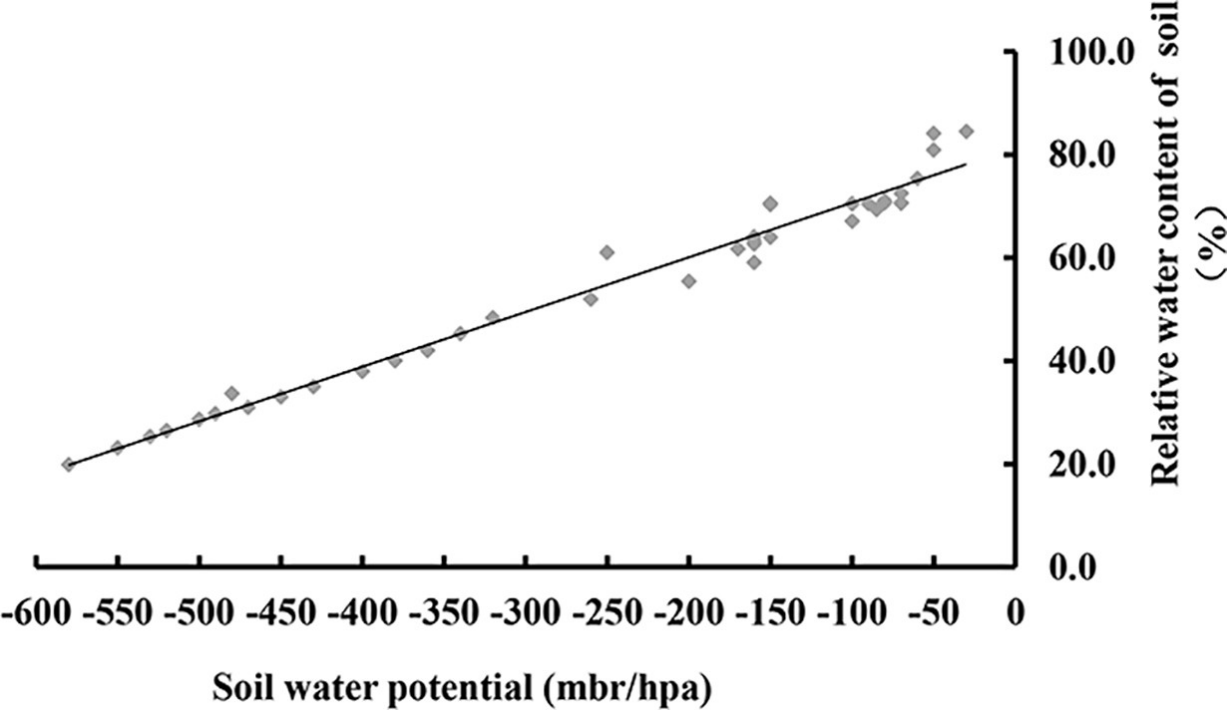
Relationship between soil water potential and soil relative water content.

Temporal data regarding the soil moisture content and soil water potential at 15, 30 and 45 cm soil depth were measured using a tensiometer (Steps, Berlin, Germany). A regression equation was calculated according to the data to describe the relationship between soil water potential and soil relative water content (Fig 2).

Irrigation amount (*I*, m^3^) was determined using the following equation: *I = r × p × h ×* θ*f × s* (q1 − q2)/η, where *r* is the soil bulk density (g cm^−3^); *p* is the soil wetting ratio; *h* is the moisture layer (0.3 m); θ*f* is the field capacity (%); *s* is the area (667m^2^); q1 and q2 are soil relative water contents with values of 80% and 60%, respectively; and η is the water use coefficient with a value of 1. According to the equation, the irrigation amount of the spring crop was 16.87m^3^ and that of the autumn crop was 15.21m^3^. Amount of water was the same for four irrigation methods in the same crop.

Storage water content (△W) was measured on the following equation:△Wx = W_1_^_^ W_0_ = θ_1_γh ^_^ θ_0_γh,Where △Wx is soil storage -water variation in 0-30cm soil layer at stage X (Before planting stage, seedling stage, rosette stage, heading stage) (m^3^);W1 is soil storage–water content at the end of stage X (m^3^); W0 is soil storage–water content at the beginning of stage X (m^3^);θ1 is soil-quality water content at the end of stage X (%);θ0 is soil-quality water content at the beginning of stage X (%);γis soil bulk density(g/cm^3^); h is soil thickness (mm).

Evaporation content was measured by micro-evaporator (PVC pipe, 5 mm thick, 100 mm diameter, 100 mm length) using the micro-lysimeter method.

Transpiration content was measured with the following equation according to the water balance method:*T* = *I* − Δ*W*–*E*; where *I* is the irrigation content (m^3^), ΔW is the storage water content (m^3^) and *E* is the evaporation content (m^3^).

### Measurement of plant height, plant weight measurement, biological yield and economic yield

Plant height from the cotyledonary node to growing point was measured by a tape. Five individual plants were measured in each treatment every 7 days. Five plants for each treatment were harvested and weighed by electronic balance. Then they were placed in a 105°C oven for 30 min followed by an 80°C oven for 48 h. The heading fresh weight was measured for economic yield and whole plant fresh weight was measured for biological yield.

### Root growth index and root distribution

Roots from five plants were sampled for each treatment down to 10 cm. The sampling is performed randomly from each treatment at harvesting time. The sampling volume is 60 in each treatment. The diameter of the core was 10 cm. The cores were washed to obtain the roots, and roots were scanned by EPSOM EXPRESSION 4990 (Seiko Epson Corporation, ChangYe, Germany). The data of root volume were analyzed using WinRHIZO (Shiya Technology, Shijiazhuang, China).

### Determination of soluble protein content, soluble sugar content, vitamin C content

For determination of soluble protein content, every leaf of lettuce was taken and mixed.1 g fresh tissue was collected and extracted with 5 ml phosphate buffer (pH 7.8) and centrifuged at 4,000*g* for 10 min at 4°C. The resultant supernatant was mixed with 5 ml of Coomassie Brilliant Blue G-250-protein reagent, and the optical density value at 595 nm was measured using a spectrophotometer UV-2102C (Honglang Company, ZhengZhou, China) [27].For determination of soluble sugar content, 0.5 g of fresh sample was ground and boiled in a water bath. Soluble sugars were measured using the traditional anthrone colorimetry method by measuring the optical density value at 630 nm [27]. For determination of Vitamin C content, 10 g of fresh tissue was ground with 2 ml of 2% oxalic acid solution, and the content of Vitamin C was measured using calibrated 2,6-dichloro indophenols [28].

### Calculation of WUE

Economic and biological WUE (EWUE and BWUE, respectively) were calculated with the following equations: EWUE = EY/*I*, BWUE = BY/*I*, where EY is the economic yield (Kg), BY is the biological yield (Kg) and *I* is the irrigation content (m^3^).

### Statistical analysis

Analysis of variance was conducted using SPSS 13.0, and data from each sampling event were analyzed separately. Mean comparisons were performed using Fisher’s least significant difference test at *P* < 0.05.

## Results

### Environmental conditions during growing seasons

S1 Table shows that average temperature in spring was 17.0°C, 19.7°C and 24.3°C in the seedling stage, rosette stage and heading stage, respectively. However, there was large variation among autumn during the three stages at 25.0°C, 19.5°C and 17.6°C, respectively. Similar variation occurred for average maximum temperature and average minimum temperature. The average humidity in spring was 83.8%, 87.2% and 83.6% in the seedling stage, rosette stage and heading stage, respectively. These values were higher than the average humidity in autumn with values of 73.8%, 77.2% and 73%, respectively. Light accumulation in the seedling stage, rosette stage and heading stage was 18.7, 22.3 and 24.7 mol m^−2^ d^−1^ in spring, respectively, and 27.6, 25.8 and 23.1 mol m^−2^ d^−1^ in autumn, respectively.

### Effects of different irrigation systems on soil bulk density, pH, EC and temperature

**Fig 3:**
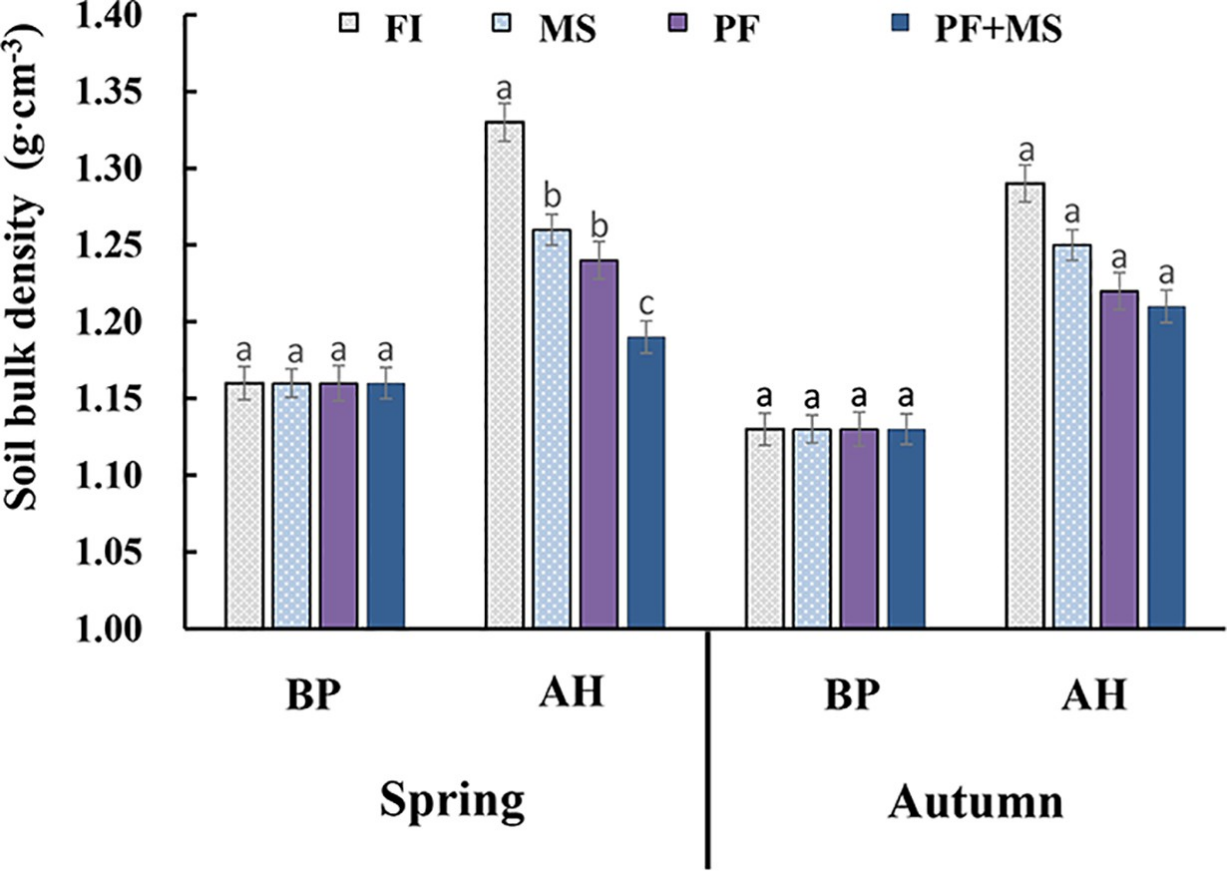
Effects of different irrigation systems on soil bulk density after harvest in spring or autumn. FI: Furrow irrigation; MS: Micro-sprinkler irrigation; PF: Plastic film mulching irrigation; PF+MS: Plastic film mulching-Micro-sprinkler irrigation (the followings are same). Values followed by different small letters are significantly different at *p* = 0.05.

Fig 3 shows that the soil bulk density was almost the same before planting in both spring and autumn. After a crop planting, soil bulk density increased in all four irrigation systems. In spring, compared with the early soil before planting, soil bulk density increased by 14.66%, 8.63%, 6.90% and 5.17% under FI, MS, PF and PF+MS, respectively. In addition, soil bulk density decreased by 5.26%, 6.77% and 10.53% under MS, PF and PF+MS, respectively, compared with FI. In autumn, the soil bulk density of FI was higher than other three irrigation systems after harvest but there was no significant difference among three irrigation systems.

S2 Table shows the variation of pH in the different soil layers before and after crop planting. The pH tended to increase after planting and with soil depth. Therefore, pH in the 20–30 cm layer was the highest of the three layers. In the spring crop, pH increased most under FI and least under PF+MS. In the 0–10 cm layer, pH increased by 0.36 in FI and increased by 0.14 in PF+MS after harvest. There were significant differences in pH among FI, PF and PF+MS and no significant difference between FI and MS after harvest. In the autumn crop, the amount of increase in soil pH was smaller than in the spring crop. The pH under FI was significantly higher than that under PF and PF+MS, indicating that micro-sprinkler and plastic film mulching could inhibit pH increase for the purpose of preventing soil alkalinization.

Soil EC represents the content of salt ions in soil solution. The higher the concentration of salt ions, the more serious the potential damage to crops. As shown in S3 Table, the EC value decreased with the increased soil depth before planting. After harvest, the EC value tended to decrease. In spring, the EC value of FI, MS, PF and PF+MS decreased by 0.26, 0.29, 0.31 and 0.33 mS cm^−1^, respectively, compared with EC before planting in the 0–10 cm soil layer. The EC value of MS, PF and PF+MS decreased by 9.68%, 16.13% and 22.58% compared with FI, respectively. The variation in EC of the 10–20 cm and 20–30 cm soil layers were similar to that at 0–10 cm. The PF+MS system produced the most significant reduction in EC in the spring crop. However, the EC value of the autumn crop was consistently higher than that of the spring crop before planting. The range of EC decrease in the autumn crop was higher than in the spring crop. After harvest, the PF system could inhibit the accumulation of salt ions in lettuce roots. Therefore, when focusing on soil protection from salinization, the effect of PF+MS was found to be optimal.

**Fig 4:**
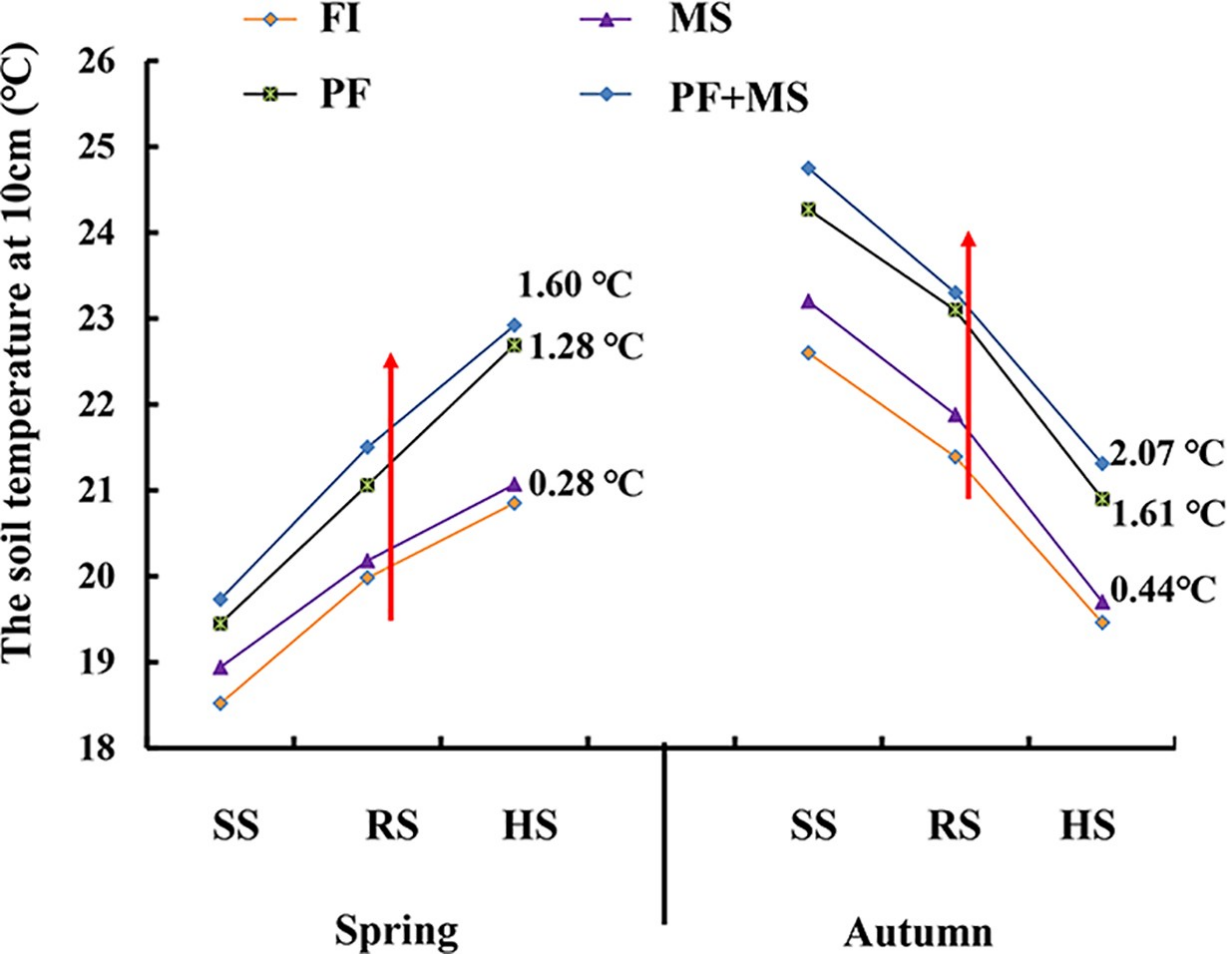
Effects of different irrigation systems on soil temperature at 10cm soil layer at SS (seedling stage),RS (rosette stage), HS (heading stage) in spring or autumn.

The variation of soil temperature during the process of growth in the 10 cm soil layer is shown in Fig 4. Soil temperature in the 10 cm soil layer gradually increased as the plant developed from the seedling stage to the rosette stage to the heading stage. In spring crop, soil temperature of four systems decreased in the order PF+MS > PF > MS > FI. The soil temperature of MS, PF, PF+MS was on average 0.28°C, 1.28°C and 1.60°C higher than FI in the three growth periods. In the autumn crop, soil temperature in the 10 cm soil layer gradually decreased as the plant developed. Soil temperature of MS, PF and PF+MS was on average 0.44°C, 1.61°C and 2.07°C higher than FI across the three growth periods. The PF+MS system had the best heat preservation effect.

### Effects of water irrigation systems on absolute amount of water distribution and water distribution through the plant

**Fig 5:**
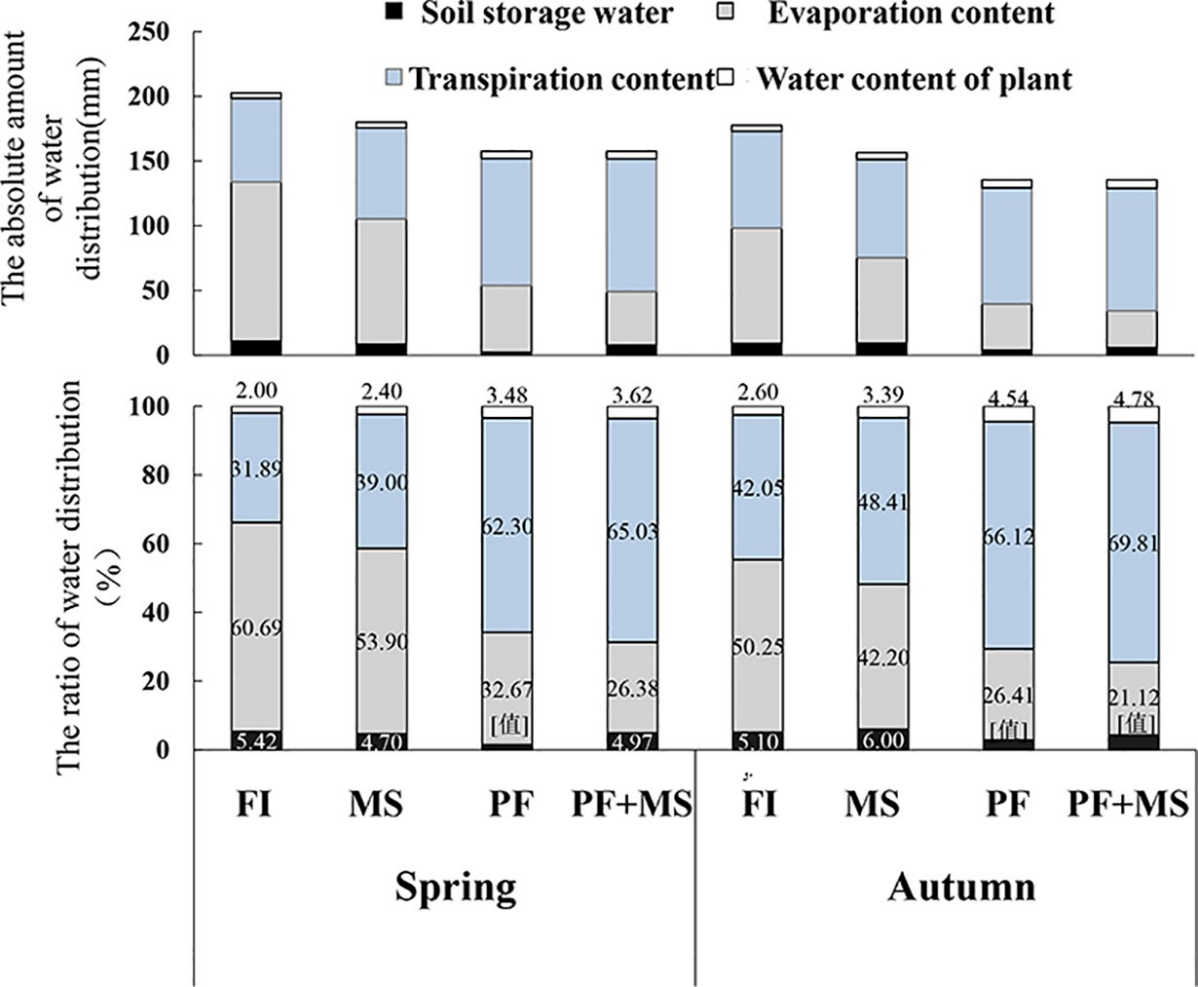
Effects of water irrigation systems on absolute amount of water distribution and water distribution through the plant.

Irrigation water distribution included soil storage water content, evaporation content, transpiration content and water content of the plant. The distribution ratio differed greatly among the different irrigation treatments. In the spring crop, as Fig 5 showed, the content of soil storage water was 5.42%, 4.70%, 1.55% and 4.97% (FI, MS, PF, PF+MS).Compared with the high soil evaporation of FI, the soil evaporation content of MS, PF, PF+MS was 53.90%, 32.67% and 26.38%,respectively. However, the soil transpiration content of FI was 31.89%,lower than MS(39.00%), PF(62.30%), PF+MS(65.03%).Data analysis showed that water content of plant at harvest increased in the order FI < MS < PF < PF+MS.

The water distribution pattern in the autumn crop was similar to that in the spring crop. The soil evaporation content in the autumn crop was lower than the spring crop; however, the soil transpiration content and water content of the plant was higher in the autumn crop than in the spring crop.

### Effects of different irrigation systems on plant height

As shown in S4 Table, plant height of lettuce was recorded from 4^th^ April to 17^th^ May in spring crop and from 19^th^ August to 30^th^ September in autumn crop. Plant height of the PF+MS and PF irrigation treatment was higher than that of FI. Plant height under MS was higher than that under FI but this difference was not significant. At harvest, plant height under MS, PF and PF+MS was 6.38%, 16.81% and 22.40% higher than under FI, respectively. The autumn is a more suitable season for the growth of lettuce. Plant height in autumn crop was higher than in the spring crop at all stages. At harvest, plant height of MS, PF and PF+MS was 5.84%, 18.84% and 19.22% higher than under FI, respectively.

### Root growth and distribution

Roots are the part of the plant that absorbs water and mineral nutrients. Root morphology and space distribution have a great influence on the absorption of water and mineral nutrients. Therefore, they could evaluate the potential ability of root. Root data regarding root growth of lettuce under different irrigation treatments are presented in S5 Table. Root length, root surface area and root density were significantly higher under the PF and PF+MS treatments, compared with those under FI. These indexes were also higher under MS than under FI but these differences were not significant. In the spring crop, root length under MS, PF and PF+MS was 16.33%, 101.51% and 117.62% higher than under FI, respectively. Root surface area under MS, PF and PF+MS was 9.69%, 20.97% and 52.69% higher than under FI, respectively. Root density under MS, PF and PF+MS was 16.53%, 101.63% and 117.81% higher than under FI, respectively. Root volume, fresh weight and dry weight decreased in the order PF+MS > PF > MS > FI. However, there was no significant difference in root volume, fresh weight and dry weight between the irrigation treatments. In the autumn crop, root physiological index was dramatically higher than in the spring crop, indicating that the soil is more suitable for root growth and absorption in autumn.

### Effects of different irrigation systems on crop weight

Data regarding weight of lettuce under different irrigation treatments are presented in S6 Table. In the spring crop, whole plant weight, aboveground weight, head weight and underground weight under PF and PF+MS were significantly higher than under FI. These indexes were also higher under MS than under FI but these differences were not significant. Whole plant fresh weight and head fresh weight are the two most important indexes to ensure balanced biological and economic production. Whole plant fresh weight under MS, PF and PF+MS was 7.22%, 36.77% and 43.20% higher than that under FI, respectively. Head weight under MS, PF and PF+MS was 10.77%, 41.49% and 46.98% higher than that under FI, respectively. In the autumn crop, physiological indexes related to crop weight decreased in the order PF+MS > PF > MS > FI. Whole plant fresh weight under MS, PF and PF+MS was 14.69%, 33.67% and 40.64% higher than under FI, respectively. Leaf head fresh weight under MS, PF and PF+MS was 18.06%, 41.48% and 58.63% higher than under FI, respectively. Whole plant dry weight under MS, PF and PF+MS was 17.23%, 40.59% and 50.31% higher than under FI, respectively. Leaf head dry weight under MS, PF and PF+MS was 16.43%, 50.85% and 54.55% higher than under FI, respectively.

### Soluble protein, soluble sugar and Vitamin C

**Fig 6:**
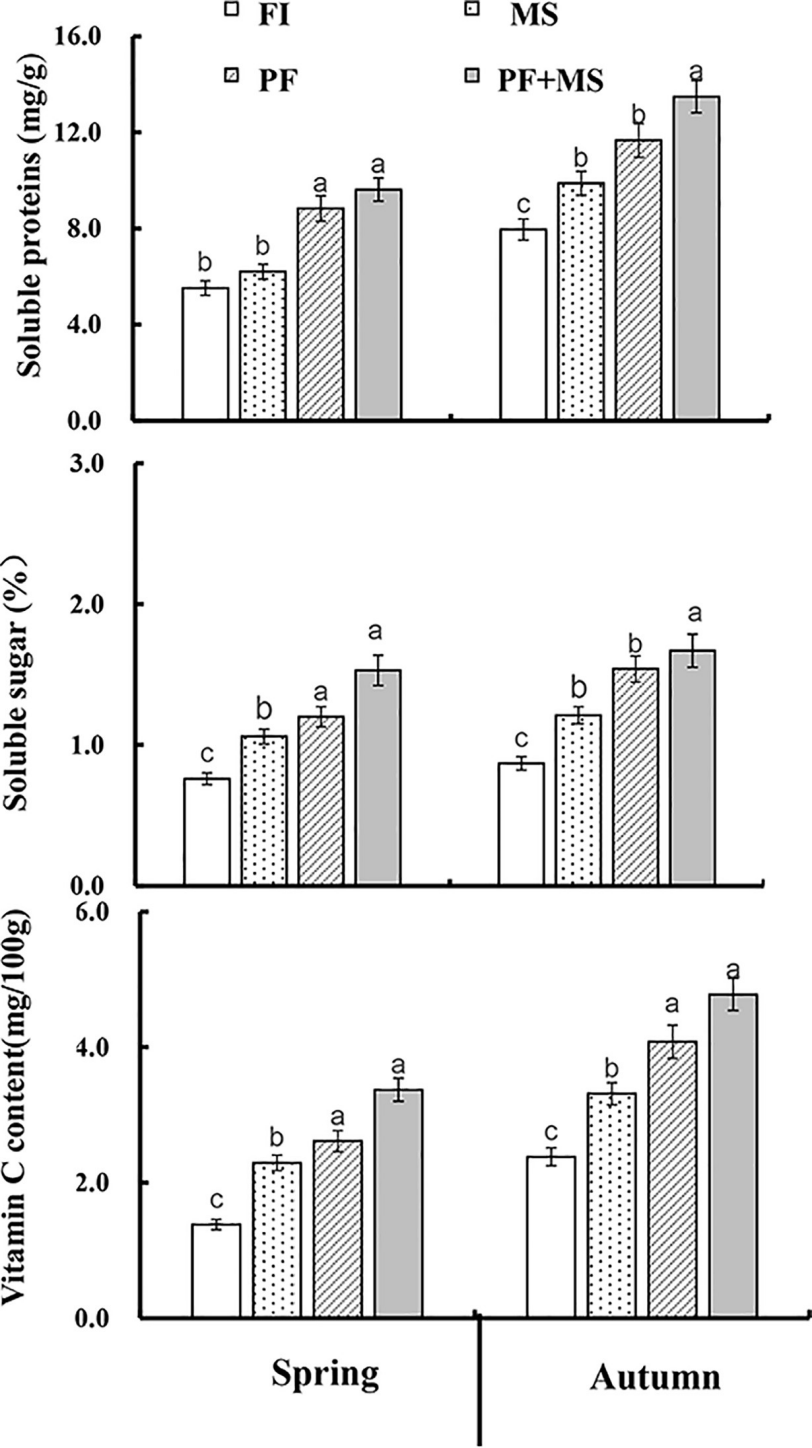
The effects of different irrigation systems on the quality of lettuce including soluble protein, soluble sugar and vitamin C content.

Lettuce quality differs in different irrigation treatment according to Fig 6.Lettuce quality is showed by soluble protein, soluble sugar and Vitamin C. In spring crop, all three indexes performed PF+MS>PF>MS>FI. Soluble protein content of MS, PF and PF+MS is 12.32%, 59.96%, 74.28% higher than FI. Soluble sugar content of MS, PF and PF+MS increased by 39.47%, 57.89%, 101.32%, as compared with FI. Data also showed that vitamin C content increased in the order PF+MS>PF>MS>FI. The change law of lettuce quality for four treatment in autumn crop is similar to spring crop. All three indexes of autumn crop are higher than spring crop. Soluble sugar content of MS, PF and PF+MS is 39.08%, 77.01%, 91.95% higher than FI in autumn crop. Soluble protein content decreased in the order FI<MS< PF<PF+MS in autumn crop. Vitamin C content increased by 39%, 71.4%, 100.8% compared with FI, indicating that PF and MS could improve lettuce quality significantly.

### Effects of different irrigation systems on nutrient absorption

Effects of different irrigation systems on nutrient absorption in differed substantially (S7 Table).Aboveground nutrient absorption was higher than underground nutrient absorption. The nutrient absorption increased in the order FI < MS < PF < PF+MS. Nutrient absorption under PF and PF+MS was significantly higher than under FI. Nutrient absorption under MS was higher than that under FI but this difference was not significant. The aboveground N content under MS, PF and PF+MS was 22.87%, 76.78% and 124.37% higher than under FI, respectively. The aboveground P content under MS, PF and PF+MS was 12.07%, 33.88% and 71.89% higher than under FI, respectively. The aboveground K content under MS, PF and PF+MS was 15.64%, 58.09% and 87.14% higher than under FI, respectively. In the autumn crop, all element contents were higher than in the spring crop, except for the underground N content.

### Yield and WUE under different irrigation systems

Yield and WUE was affected by the different irrigation systems (S8 Table). Lettuce yield is the most important index for evaluating water-saving system. In the spring crop, biological yield and economic yield decreased in the order PF+MS > PF > MS > FI. Biological yield of MS, PF and PF+MS was 7.22%, 36.77% and 43.20% higher than under FI, respectively. Economic yield was 10.71%, 41.49% and 46.97% higher than under FI, respectively. The BWUE and EWUE of MS, PF and PF+MS were prominently higher than those under FI, respectively. The BWUE of FI, MS, PF and PF+MS was 20.93, 25.24, 36.81 and 38.54 kg m^−3^, respectively, producing values 20.59%, 75.87% and 84.14% higher than those under FI, respectively. The EWUE of FI, MS, PF and PF+MS was 17.06, 21.31, 31.11 and 32.31 kg m^−3^, respectively, producing values 24.91%, 82.36% and 89.39% higher than those under FI, respectively. However, in the autumn crop, the biological and economic yield of each irrigation system was higher than in the spring crop. Subsequently, the BWUE and EWUE of the autumn crop were higher than those of the spring crop. In spring crop, the amount of saved water is 30m^3^ using the PF+MS method compared with FI.

## Discussion

### Effect of water-saving irrigation systems on soil environment

Light is one of the most important environmental factors for crop growth and development [29].Lettuce is suited to a cool climate; an appropriate growing temperature for lettuce is 15–20°C [30].Under the condition of low temperature and high humidity, lettuce is vulnerable to downy mildew, which reduces lettuce quality and yield [31].In autumn, temperature is similar to that in spring. In addition, humidity is lower and light accumulation is higher than that in spring. So, plant height in autumn is higher than that in spring. Biological yield of four treatments in autumn is higher than that in spring, respectively. Lettuce grows well under autumn crop than under spring crop.

Soil bulk density is a sensitive indicator of soil compaction and larger number often indicates barren soil [32]. The results from this study demonstrate that soil bulk density was highest under FI and lowest under PF+MS after crop planting, indicating that the combined plastic film and micro-sprinkler system could maintain good soil physical characteristics and inhibit the increase of soil bulk density. As a consequence, this soil permeability is increased, which is favorable for lettuce growth. Soil pH is a major controlling factor in soils and is influenced by many chemical processes. It particularly affects plant nutrient availability by controlling the chemical forms of the different nutrients and affecting the chemical reactions they undergo. The optimum pH range for most plants is from 6.5 to 7.5; however, many plants have adapted to survive at pH values outside this range [33]. As shown in S2 Table, there was an increasing pH trend over soil depth in both the spring crop and autumn crop in the current study. The pH value under the plastic film mulching treatments was lower than under the non-plastic film mulching treatments. This may be because soil temperature and humidity increased and promoted the release of N nutrients during the early stages under the plastic film mulching treatments. The alkali-hydrolyzable N content may have subsequently improved and a large concentration of nitric nitrogen led to the decrease of soil pH [34, 35]. The results presented in S3 Table indicate that plastic film mulching treatment had a lower EC than non-plastic film mulching. Similarly, some previous studies have shown that non-mulched treatments had higher EC than mulched treatments [36–38], however, the other found the opposite [39, 40]. These contradictions may occur because the different climatic conditions lead to different soil surface evaporation, especially during the early plant growth stages, resulting in different amounts of utilization of precipitation [41].

### Effect of water-saving irrigation systems on water distribution

Many studies have shown that over-irrigation results in water losses, increased vulnerability to diseases and environmental pollution arising from fertilizer loss [9, 42]. Previous studies have conducted a large number of analyses focusing on irrigation water distribution and theory. A study on water-saving systems indicated that water is mainly distributed in the 0–60 cm soil layer. Drip irrigation and micro-sprinkler irrigation are more water-saving than pipe irrigation. The amount of water consumed by drip irrigation and micro-sprinkler irrigation has been found to be 12.49% and 1.52% less than pipe irrigation, respectively. In addition, micro-irrigation could save water compared with flood irrigation under the reduction in water losses from evaporation and deep percolation as well as savings during water delivery [43, 44]. Plastic film mulch could reduce water loss from the soil [45], and increases the soil water content in the upper soil layer [46, 47]. In the current study, the water distribution pattern changed after plastic film mulch treatment (Fig 5). The air temperature at the soil surface was higher under the plastic film mulch treatments than under the non-plastic film mulch treatments. Vapor pressure of the plastic film mulch treatment is lower and affects water exchange between air and soil. As a result, the evaporation water content of the soil reduces and the proportion of transpiration water content in the plant improves. The rate of transpiration is also influenced by the evaporative demand of the atmosphere surrounding the leaf such as boundary layer conductance, humidity, temperature, wind and incident sunlight [48]. Soil water supply and soil temperature can influence stomatal opening, and thus the transpiration rate [49]. Plant transpiration can provide water and nutrients for plant photosynthesis and plant growth. Irrigation water distributed for plant transpiration is effective water. The increasing percentage of transpiration water could improve plant production and WUE. Several previous experiments have shown that drip irrigation, micro-irrigation and mulching are able to reduce soil water evaporation loss to some extent [50–52].

### Effect of water-saving irrigation systems on root growth, plant growth and WUE

The soil environment produced under different water-saving irrigation systems varies. In particular, soil surface water content and soil temperature increase after plastic film mulching. As a result, roots thrive in the soil and promote crop absorption of water and mineral elements [53–55]. Previous research has indicated that crop root length, root density, root fresh weight and dry weight were significantly higher under plastic film mulching treatments than under non-plastic film mulching treatments [56, 57]. In the current study, root indexes such as root length, root volume and root density under the PF and PF+MS were significantly higher than under FI in the 0–10 cm soil layer (S5 Table).The root indexes were higher under MS than under FI but this difference was not significant. In addition, Zhang et al. (2009) revealed that most of the root growth of winter wheat was in the uppermost soil profile [58]. Moreover, the absorption of water stored in the 15–30 cm soil layer had greater effects on crop growth than that of deeper root system [59].

Use of optimized irrigation systems and efficient irrigation methods has the potential to increase WUE and save irrigation water. As shown in S8 Table, BWUE and EWUE were highest under the PF+MS irrigation method of the four irrigation systems. The BWUE and EWUE under PF+MS was 84.14% and 89.39%, respectively higher than under FI. Similarly, WUE of potato under the plastic-film mulch treatment was shown to be significantly higher than WUE under the non-mulch treatment [60].The increased yield and WUE with plastic-film mulch reported for the current study were consistent with previous reports [20, 61]. About financial benefit, it costs about $210, $250, $450 under PF, MS, PF+MS treatment per acre. Financial benefit under PF+MS treatment is 46.97% higher than that under FI.

## Conclusion

Micro-sprinkler and plastic film mulch were both found to prevent the increase of soil bulk density and pH, maintain good physical characteristics, increase the transformation of fertilizer and improve the soil environment. In addition, micro-sprinkler and plastic film could decrease invalid evaporation, reduce the irrigation amount and increase the percentage of plant transpiration water. Micro-sprinkler and plastic film mulching could change the root distribution, promote root growth, increase plant height, improve lettuce quality and promote the absorption of mineral elements. In both the spring crop and autumn crop, yield and WUE under the different irrigation treatments decreased in the order PF+MS > PF > MS > FI. This indicates that the micro-sprinkler and plastic film irrigation methods both have the potential to increase lettuce yield and WUE and the optimum effect occurs under the combination of plastic film mulching with micro-sprinkler irrigation. This study pushed practical application in lettuce greenhouse agriculture in future research.

## Supporting information

S1 Table Environmental conditions of different growth stage in the greenhouse. Environmental conditions of different growth stage in the greenhouse. SS: seedling stage; RS: rosette stage; HS: heading stage; AHT: average highest temperature; ALT: average lowest temperature; AT: average temperature; AH: average humidity; LA: light accumulation.
(PDF)

S2 Table The effects of different irrigation systems on soil pH in three soil layers. (PDF)

S3 Table The effects of irrigation systems on soil EC in three soil layer. (PDF)

S4 Table The effects of different irrigation systems on plant height at the time-course developmental stage in spring and autumn. (PDF)

S5 Table Effects of different irrigation systems on the distribution and growth of root (0~10cm). RL: root length; RSA: root surface area; RV: root volume; RD:root density; FW:fresh weight; DW:dry weight.
(PDF)

S6 Table Effects of different irrigation systems on whole plant weight, aboveground and underground weight (g/plant). (PDF)

S7 Table Effects of different irrigation systems on nutrient absorption (g/ plantD· W). (PDF)

S8 Table Effects of different irrigation systems on yield and water use efficiency. (PDF)

S1 Data The original data for Figures and Tables. (RAR)

## Abbreviations

FI: Furrow Irrigation
MS: Micro-sprinkler Irrigation
PF: Plastic Film mulching- Irrigation
PF+MS: Plastic Film mulching-Micro-sprinkler Irrigation
AHT: Average highest temperature
ALT: Average lowest temperature
AT: Average temperature
AH: Average humidity
LA: Light accumulation
BP: Before Planting
AH: After Harvesting
SS: Seedling Stage
RS: Rosette Stage
HS: Heading Stage
E: The Content of Evaporation
T: The Content of Transpiration
RL: Root length
RSA: Root Surface Area
RV: Root volume
RD: Root density
FW: Fresh weight
DW: Dry weight
SC: Spring crop
AC: Autumn crop
BY: Biological Yield
EY: Economic Yield
BWUE: Water Use Efficiency of Biological Yield
EWUE: Water Use Efficiency of Economic Yield
