# Supplementary material for: Assessing the performance of different irrigation systems on lettuce (Lactuca sativa L.) in the greenhouse
Source: PLoS One. 2019 Feb 4;14(2):e0209329. doi: 10.1371/journal.pone.0209329 (PMC6361420; doi:10.1371/journal.pone.0209329)
Supplement: S1 Table — Environmental conditions of different growth stage in the greenhouse. SS: seedling stage; RS: rosette stage; HS: heading stage; AHT: average highest temperature; ALT: average lowest temperature; AT: average temperature; AH: average humidity; LA: light accumulation. (PDF) [file pone.0209329.s001.pdf]

S1 Table . Environmental conditions of different growth stage in the greenhouse. SS: seedling stage; RS: rosette stage; HS: heading stage; AHT: average highest temperature; ALT: average lowest temperature; AT: average temperature; AH: average humidity; LA: light accumulation

Period	Growth Stage	AHT (℃)	ALT (℃)	AT(℃)	AH(%)	LA (molm^{-2} d^{-1})
Spring	SS	24.9	12.0	17.0	83.8	18.7
	RS	27.5	14.0	19.7	87.2	22.3
	HS	31.7	18.2	24.3	83.6	24.7
Autumn	SS	32.3	19.1	25.0	73.8	27.6
	RS	29.3	15.6	19.5	77.2	25.8
	HS	25.8	12.2	17.6	73.0	23.1
