# Supplementary material for: Assessing the performance of different irrigation systems on lettuce (Lactuca sativa L.) in the greenhouse
Source: PLoS One. 2019 Feb 4;14(2):e0209329. doi: 10.1371/journal.pone.0209329 (PMC6361420; doi:10.1371/journal.pone.0209329)
Supplement: S2 Table — (PDF) [file pone.0209329.s002.pdf]

S2 Table . The effects of different irrigation systems on soil pH in three soil layers.

Soil layer	Treatment	Spring		Autumn	
		BP	AH	BP	AH
0～10cm	FI	7.27a	7.63a	7.38a	7.57a
	MS	7.27a	7.61a	7.38a	7.51a
	PF	7.27a	7.49b	7.38a	7.45b
	PF+MS	7.27a	7.44b	7.38a	7.42b
10～20cm	FI	7.34a	7.67a	7.43a	7.67a
	MS	7.34a	7.63a	7.43a	7.62a
	PF	7.34a	7.50b	7.43a	7.55b
	PF+MS	7.34a	7.47b	7.43a	7.54b
20～30cm	FI	7.46a	7.79a	7.47a	7.75a
	MS	7.46a	7.74a	7.47a	7.69a
	PF	7.46a	7.65b	7.47a	7.62b
	PF+MS	7.46a	7.59b	7.47a	7.60b

Note: Under the same column, values followed with the same letter was not significant at $P = 0.05$
