# Supplementary material for: Assessing the performance of different irrigation systems on lettuce (Lactuca sativa L.) in the greenhouse
Source: PLoS One. 2019 Feb 4;14(2):e0209329. doi: 10.1371/journal.pone.0209329 (PMC6361420; doi:10.1371/journal.pone.0209329)
Supplement: S3 Table — (PDF) [file pone.0209329.s003.pdf]

S3 Table. The effects of irrigation systems on soil EC in three soil layer.

Soil layer	Treatment	Spring		Autumn	
		BP	AH	BP	AH
0～10cm	FI	0.57a	0.31a	0.63a	0.41a
	MS	0.57a	0.28b	0.63a	0.35b
	PF	0.57a	0.26b	0.63a	0.31b
	PF+MS	0.57a	0.24b	0.63a	0.28c
10～20cm	FI	0.46a	0.28a	0.55a	0.32a
	MS	0.46a	0.25a	0.55a	0.28a
	PF	0.46a	0.22b	0.55a	0.25b
	PF+MS	0.46a	0.20b	0.55a	0.24b
20～30cm	FI	0.36a	0.27a	0.44a	0.36a
	MS	0.36a	0.25a	0.44a	0.30b
	PF	0.36a	0.23b	0.44a	0.27b
	PF+MS	0.36a	0.21b	0.44a	0.23c

Note: Under the same column, values followed with the same letter was not significant at *P* = 0.05
