# Supplementary material for: Assessing the performance of different irrigation systems on lettuce (Lactuca sativa L.) in the greenhouse
Source: PLoS One. 2019 Feb 4;14(2):e0209329. doi: 10.1371/journal.pone.0209329 (PMC6361420; doi:10.1371/journal.pone.0209329)
Supplement: S4 Table — (PDF) [file pone.0209329.s004.pdf]

S4 Table. The effects of different irrigation systems on plant height at the time-course developmental stage in spring and autumn.

	Treatment	4th April	11th April	18th April	25th April	2th May	9th May	17th May
Spring (cm)	FI	7.95a	10.13b	12.14b	15.81b	17.25c	18.23c	20.68b
	MS	8.04a	10.77b	12.99b	16.27b	18.57c	21.43b	22.09b
	PF	8.19a	12.43a	15.63a	17.29a	20.59b	22.67b	24.86a
	PF+MS	8.23a	12.95a	16.48a	19.45a	21.47a	24.83a	26.65a
		19th August	26th August	2th September	9th September	16th September	23th September	30th September
Autumn (cm)	FI	7.99a	11.66c	13.16b	16.01b	18.01c	20.46c	22.07b
	MS	8.15a	12.69b	14.27b	16.43b	19.25b	22.15b	23.44b
	PF	8.29a	13.39b	15.76a	18.27a	22.15b	23.16a	26.23a
	PF+MS	8.31a	13.87a	16.98a	18.89a	24.45a	26.23a	27.32a

Note: Under the same column, values followed with the same letter was not significant at *P* = 0.05
