# Supplementary material for: Assessing the performance of different irrigation systems on lettuce (Lactuca sativa L.) in the greenhouse
Source: PLoS One. 2019 Feb 4;14(2):e0209329. doi: 10.1371/journal.pone.0209329 (PMC6361420; doi:10.1371/journal.pone.0209329)
Supplement: S5 Table — RL: root length; RSA: root surface area; RV: root volume; RD:root density; FW:fresh weight; DW:dry weight. (PDF) [file pone.0209329.s005.pdf]

S5 Table. Effects of different irrigation systems on the distribution and growth of root (0～10cm).RL: root length; RSA: root surface area; RV: root volume; RD:root density; FW:fresh weight; DW:dry weight.

	Treatment	RL (cm/plant)	RSA (cm^2/plant)	RV (cm^3/plant)	RD ($cm \cdot cm^{-3}$)	FW (g/plant)	DW (g/plant)
Spring	FI	674.75b	135.09b	3.283a	0.859b	8.247a	0.685a
	MS	784.97b	148.18b	3.423a	1.001b	9.207a	0.712a
	PF	1359.67a	163.42a	3.668a	1.732a	9.481a	0.739a
	PF+MS	1468.40a	206.27a	3.725a	1.871a	9.617a	0.797a
Autumn	FI	703.251c	164.65b	3.417a	0.896c	11.616b	0.830c
	MS	1172.11b	183.01b	3.528a	1.493b	12.426b	1.044b
	PF	1421.88a	251.54a	3.629a	1.811a	15.894a	1.075a
	PF+MS	1681.38a	259.65a	4.039a	2.142a	18.894a	1.116a

Note: Under the same column, values followed with the same letter was not significant at *P* = 0.05
