# Supplementary material for: Assessing the performance of different irrigation systems on lettuce (Lactuca sativa L.) in the greenhouse
Source: PLoS One. 2019 Feb 4;14(2):e0209329. doi: 10.1371/journal.pone.0209329 (PMC6361420; doi:10.1371/journal.pone.0209329)
Supplement: S6 Table — (PDF) [file pone.0209329.s006.pdf]

S6 Table . Effects of different irrigation systems on whole plant weight, aboveground and underground weight (g/plant).

	Treatment	Whole plant		Aboveground		Leaf heading		Underground	
		FW	DW	FW	DW	FW	DW	FW	DW
SC	FI	586.84b	24.751b	563.50b	22.239b	479.47b	17.091c	23.34c	2.512a
	MS	629.22b	29.413b	600.59b	26.732b	531.11b	20.461b	28.63b	2.681a
	PF	802.65a	41.512a	772.57a	38.500a	678.42a	27.141a	30.08a	3.012a
	PF+MS	840.37a	47.280a	809.26a	43.784a	704.71a	30.032a	31.11a	3.496a
AC	FI	672.32d	32.690c	632.29c	30.643c	486.47d	22.197c	40.03c	2.053b
	MS	771.10c	38.323b	729.71b	35.412b	574.34c	25.843b	41.39b	2.915a
	PF	898.71b	45.959a	847.52a	42.952a	688.27b	30.937b	51.19a	3.017a
	PF+MS	945.58a	49.135a	893.49a	45.825a	771.71a	34.305a	52.09a	3.314a

Note: Under the same column, values followed with the same letter was not significant at *P* = 0.05
