# Supplementary material for: Assessing the performance of different irrigation systems on lettuce (Lactuca sativa L.) in the greenhouse
Source: PLoS One. 2019 Feb 4;14(2):e0209329. doi: 10.1371/journal.pone.0209329 (PMC6361420; doi:10.1371/journal.pone.0209329)
Supplement: S7 Table — (PDF) [file pone.0209329.s007.pdf]

S7 Table . Effects of different irrigation systems on nutrient absorption (g/ plant ·DW)

	Treatment	Aboveground			Underground		
		N	P	K	N	P	K
Spring	FI	1.700b	0.323b	0.306b	0.101b	0.026c	0.074b
	MS	2.227b	0.426b	0.404b	0.124b	0.029c	0.085b
	PF	3.579a	0.726a	0.774a	0.179a	0.035b	0.116a
	PF+MS	4.306a	0.882a	1.041a	0.227a	0.045a	0.138a
Autumn	FI	2.380b	0.544b	0.528b	0.095c	0.029c	0.067c
	MS	3.100b	0.701b	0.681b	0.157b	0.042b	0.110b
	PF	3.994a	1.010a	0.954a	0.192b	0.044b	0.123a
	PF+MS	4.532a	1.240a	1.120a	0.250a	0.052a	0.141a

Note: Under the same column, values followed with the same letter was not significant at *P* = 0.05
