# Supplementary material for: Assessing the performance of different irrigation systems on lettuce (Lactuca sativa L.) in the greenhouse
Source: PLoS One. 2019 Feb 4;14(2):e0209329. doi: 10.1371/journal.pone.0209329 (PMC6361420; doi:10.1371/journal.pone.0209329)
Supplement: S8 Table — (PDF) [file pone.0209329.s008.pdf]

S8 Table . Effects of different irrigation systems on yield and water use efficiency.

	Treatment	Yield		Water use efficiency	
		BY (kg)	EY (kg)	B WUE (kg/m^3)	E WUE (kg/m^3)
Spring	FI	2826.79b	2309.63b	20.93c	17.06c
	MS	3030.95b	2558.34b	25.24b	21.31b
	PF	3866.34a	3267.96a	36.81a	31.11a
	PF+MS	4048.04a	3394.54a	38.54a	32.31a
Autumn	FI	3238.57c	2343.33d	27.11c	19.62c
	MS	3714.39b	2766.60c	35.31b	26.30b
	PF	4329.09a	3315.40b	47.90a	36.68a
	PF+MS	4554.86a	3717.33a	50.40a	41.13a

Note: Under the same column, values followed with the same letter was not significant at $P = 0.05$
